# Testing the cognitive effects of tadalafil. Neuropsychological secondary outcomes from the PASTIS trial

**DOI:** 10.1016/j.cccb.2023.100187

**Published:** 2023-09-26

**Authors:** Mathilde MH Pauls, Jessica Fish, Lauren R Binnie, Philip Benjamin, Shai Betteridge, Brian Clarke, Mohani-Preet K Dhillon, Rita Ghatala, Fearghal AH Hainsworth, Franklyn A Howe, Usman Khan, Christina Kruuse, Jeremy B Madigan, Barry Moynihan, Bhavini Patel, Anthony C Pereira, Egill Rostrup, Anan BY Shtaya, Catherine A Spilling, Sarah Trippier, Rebecca Williams, Robin Young, Thomas R Barrick, Jeremy D Isaacs, Atticus H Hainsworth

**Affiliations:** a Molecular & Clinical Sciences Research Institute, St George's University of London, UK; b Department of Neurology, St George's University Hospitals NHS Foundation Trust, London, UK; c Neuropsychology, St George's University Hospitals NHS Foundation Trust, London, UK; d School of Health & Wellbeing, University of Glasgow, UK; e Neuroradiology, St George's University Hospitals NHS Foundation Trust, London, UK; f South London Stroke Research Network, London, UK; g Department of Neurology and Neurovascular Research Unit, Herlev Gentofte Hospital, Denmark; h Department of Medicine, Royal College of Surgeons in Ireland, Beaumont Hospital, Dublin, Ireland; i Mental Health Centre, University of Copenhagen, Glostrup, Denmark; j Robertson Centre for Biostatistics, University of Glasgow, UK

**Keywords:** Clinical trials, Tadalafil, Pde5, Small vessel disease, Vascular cognitive impairment, Vascular dementia, Cognitive testing

## Abstract

Cerebral small vessel disease (SVD) is a major cause of cognitive impairment in older people. As secondary endpoints in a phase-2 randomised clinical trial, we tested the effects of single administration of a widely-used PDE5 inhibitor, tadalafil, on cognitive performance in older people with SVD. In a double-blinded, placebo-controlled, cross-over trial, participants received tadalafil (20 mg) and placebo on two visits ≥ 7 days apart (randomised to order of treatment). The Montreal Cognitive Assessment (MOCA) was administered at baseline, alongside a measure to estimate optimal intellectual ability (Test of Premorbid Function). Then, before and after treatment, a battery of neuropsychological tests was administered, assessing aspects of attention, information processing speed, working memory and executive function. Sixty-five participants were recruited and 55 completed the protocol (*N* = 55, age: 66.8 (8.6) years, range 52–87; 15/40 female/male). Median MOCA score was 26 (IQR: 23, 27], range 15–30). No significant treatment effects were seen in any of the neuropsychological tests. There was a trend towards improved performance on Digit Span Forward (treatment effect 0.37, C.I. 0.01, 0.72; *P* = 0.0521). We did not identify significant treatment effects of single-administration tadalafil on neuropsychological performance in older people with SVD. The trend observed on Digit Span Forward may help to inform future studies.

**Clinical trial registration:**

http://www.clinicaltrials.gov. Unique identifier: NCT00123456, https://eudract.ema.europa.eu. Unique identifier: 2015–001,235–20NCT00123456.

## Highlights

• More drugs are needed for vascular causes of dementia.
• Single-administration tadalafil did not enhance cognition in people with small vessel disease.
• Chronic tadalafil treatment may have cognitive effects.

## 1 Introduction

Vascular cognitive impairment (VCI) is a major contributor to dementia in older adults worldwide [1]. A widespread cause of VCI is cerebral small vessel disease (SVD) [2,3]. With few treatment options for SVD or VCI, re-purposing existing drugs is an attractive approach [4].

As nitric oxide-cGMP signaling participates in cerebrovascular function, as well as synaptic function, we reasoned that augmenting this signaling pathway could influence SVD and VCI. The cytoplasmic enzyme phosphodiesterase-5 (PDE5) degrades cGMP and potent, selective PDE5 inhibitors (PDE5i) are available. The PDE5i drugs sildenafil, vardenafil and tadalafil are in clinical use for erectile dysfunction and pulmonary arterial hypertension. PDE5 is present in vascular myocytes within human brain [5] and also in human brain neurons [6]. In a phase-2 clinical trial, we tested whether single administration of tadalafil increased cerebral blood flow (CBF) in older people with symptomatic SVD [7]. Tadalafil was chosen because it has a relatively-long plasma half-life (16 h in healthy adults) [8,9], with evidence of brain penetration in rodents and primates [10,11].

Neuropsychological performance was specified prospectively as a secondary outcome in the PASTIS trial [7]. Some prior data from pre-clinical paradigms suggested a cognitive effect of PDE5i treatment, some with rapid actions [11], [12], [13], [14], [15]. Acute dosing of rats with sildenafil improved performance in a test of executive function [14] and reversed the impairment in learning produced by the nitric oxide synthase inhibitor l-NAME [15]. Cognitive benefits of tadalafil might be hypothesized, at least on clinical tests in humans [16], as more likely to accrue over time by preventing decline in cognitive function, rather than after a single administration. Nonetheless, we included change in neuropsychological tests as secondary endpoints.

We performed a double blinded, randomized clinical trial, testing the PDE5i tadalafil for effects on cerebral blood flow (CBF). The trial was neutral and we recently reported the primary outcomes [17]. Neuropsychological test data were secondary outcomes. Here we present neuropsychological data from the PASTIS trial, testing whether a single administration of tadalafil changes cognitive performance.

## 2 Materials and methods

This trial was preregistered at http://www.clinicaltrials.gov (Unique identifier: NCT00123456) and https://eudract.ema.europa.eu (Unique identifier: 2015–001,235–20NCT00123456). The data supporting this report are available from the corresponding author upon reasonable request. We will deposit the data in the DementiasplatformUK porta**l (**Home - DPUK Data Portal (dementiasplatform.uk).

### 2.1 Trial design, randomization and endpoints

**Fig. 1:**
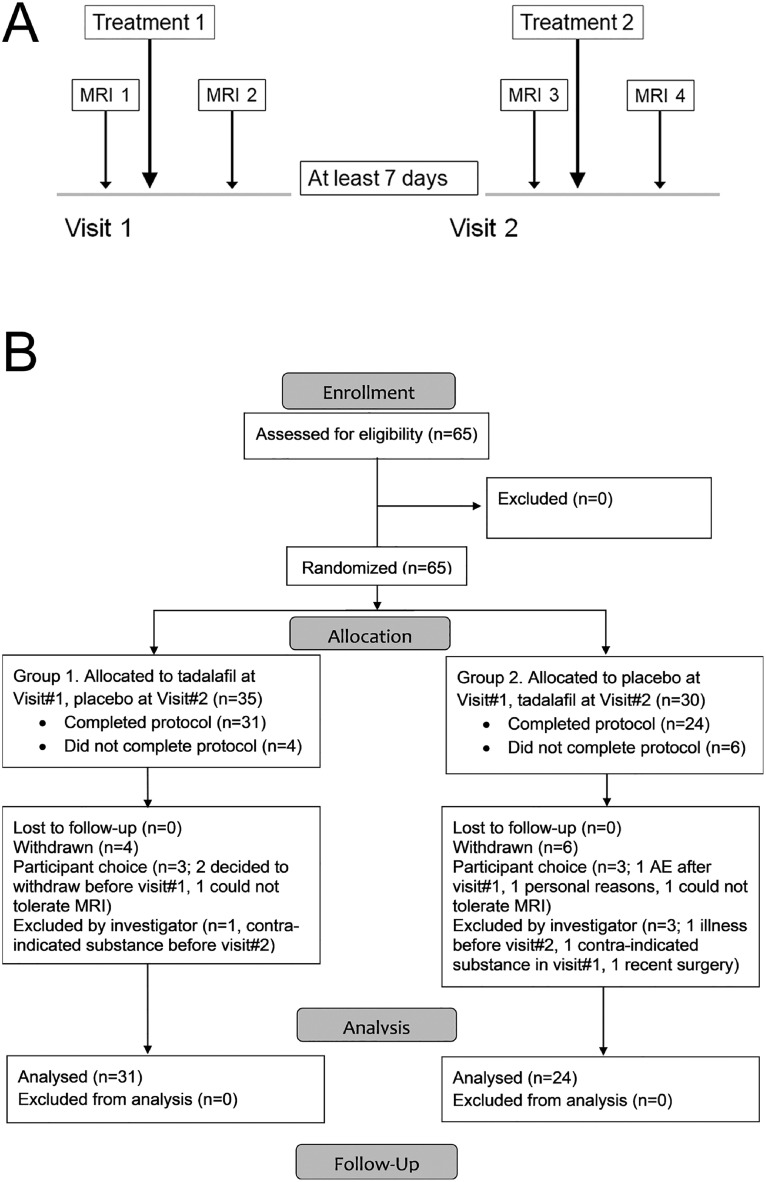
Trial design and recruitment.

The trial received ethical approval from the UK National Research Ethics Service (REC reference: 15/LO/0714). Within the UK the National Research Ethics Service, part of the NHS Health Research Authority (https://www.hra.nhs.uk/) enacts the principles of the Declaration of Helsinki (and subsequent amendments; World Medical Association) for medical research involving human subjects. Written informed consent was obtained from all participants or their next of kin. Participants were enrolled by members of the trial team and randomised to order of treatment (tadalafil 20 mg, placebo; oral administration). The randomization list was generated in advance by Sharp Clinical Services, Crickhowell, Powys, UK. Each participant received on two separate occasions, visit#1 and visit#2, a placebo dose and a tadalafil 20 mg dose which were identical in size, shape, weight and color. Two study visits were performed at least 7 days apart, with blood pressure measurement, MRI scanning and a battery of cognitive tests up to 3 h before and 3–5 h after dosing (see Fig. 1A). Participants, care providers and those assessing outcomes were all blind to treatment allocation. The primary endpoint was change in subcortical CBF. Neuropsychological tests were used as secondary endpoints. The following cognitive instruments were specified as secondary outcomes [7,18]: CANTAB simple and choice reaction time (RT); speed of information processing (adjusted and total); digit span forward and backward; semantic fluency. As the protocol entailed two visits, each with a pre-dosing and post-dosing assessment, we employed tests with four alternative versions to avoid learning effects.

Fig. 1. Design and recruitment of the PASTIS trial [17]. A, protocol for study visits. Participants were randomized to order of treatment. Group 1 received tadalafil on visit #1 and placebo on visit #2, Group 2 vice versa. B, CONSORT diagram for the PASTIS trial.

The trial commenced 4th September 2015. Participants were recruited from St George's Hospital and local Participant Identification Centres. All visits, data management and trial coordination were performed at the St George's site. The trial ended when the pre-determined recruitment target was met (25 January 2018). See CONSORT flow diagram, Fig. 1B.

### 2.2 Study population

All data were from older adults without a known diagnosis of dementia, with radiological and clinical evidence of symptomatic SVD. People with a known diagnosis of dementia were excluded, based on existing clinical records at the time of recruitment. For further details on Inclusion/Exclusion criteria, see the Supplementary file, also the study protocol [7].

### 2.3 Study assessments

In the screening visit (“Visit 0″) informed consent was documented and education level and Montreal Cognitive Assessment (MoCA) scores were recorded, along with estimated premorbid IQ as measured by the Test of Premorbid Function (TOPF). In study visits (Visit#1, Visit#2) participants underwent blood pressure measurements, brain MRI and a neuropsychological test battery [7]. The battery of neuropsychological tests was designed to assess aspects of attention, information processing speed, working memory and executive function. As the trial protocol entailed two visits, each with a pre- and post-dosing assessment, we employed cognitive tests with four alternative versions to avoid a learning effect, administered in a random order. All cognitive tests were administered by an experienced researcher who had received training in administering the study battery from a Consultant Clinical Neuropsychologist (SB), who also provided ongoing supervision.

The tests administered were: CANTAB® Reaction Time subtest; Speed of Information Processing (SOIP) subtest of the BMIPB (Brain Injury Rehabilitation Trust Memory and Information Processing Battery; Digit Span Forward (DSF) and Digit Span Backward (DSB), from Repeatable Battery for the Assessment of Neuropsychological Status (RBANS); Semantic fluency (also from RBANS). Aside from the TOPF and the MOCA (which were baseline-only measures) the test battery was such that it was suitable for administration to any fluent speaker of English. Hence no modifications of the standardised administration procedures for non-native English speakers were applied.

At the end of each study visit, and at least 3 h post dosing, two blood samples (5 ml) were taken for full blood count and analysis of tadalafil concentration. For further details, see the Supplementary file.

### 2.4 Statistical analysis

All analyses were based on the intention-to-treat principle (i.e. participants were analyzed according to randomized treatment group regardless of whether they received the intended treatment). Change within each treatment group was analysed using paired sample t-tests. Treatment effects were defined as [(after tadalafil-before tadalafil)-(after placebo-before placebo)]. Treatment effects on primary and secondary outcomes were analysed using linear mixed effects models with Wald confidence intervals, with fixed effects of baseline value, treatment, visit and random effect of subject. Models were not corrected for age, blood pressure or full blood count. No imputation or other missing data approaches were used in the analysis plan. Analysis was conducted using R v.3.4.1 with the lme4 and lmerTest packages (https://www.R-project.org/). No corrections were made for multiple comparisons. *p* < 0.05 was considered significant.

## 3 Results

Sixty-five individuals gave consent and were randomized and 55 completed the protocol (Fig. 1B). There were no clinically-meaningful demographic differences between those randomized and those who completed the protocol (Table 1).

**Table 1:** Participant demographics for the study cohort.

	Participants who consented and were randomised (*N* = 65)			Participants who completed the protocol (*N* = 55)		
Variable	All	Group 1. tadalafil followed by placebo	Group 2. placebo followed by tadalafil	All	Group 1. tadalafil followed by placebo	Group 2. placebo followed by tadalafil
N	65	33	32	55	30	25
Age (y)	66.7 (8.7)	65.5 (9.0)	68.0 (8.4)	66.8 (8.6)	65.9 (8.9)	67.9 (8.4)
Age range (y)	52, 87	52, 83	53, 87	52, 87	52, 83	53, 87
Female/Male	19/46	5/28	14/18	15/40	5/25	10/15
MoCA score	25.4 (3.4)	25.4 (3.6)	25.4 (3.3)	25.1 (3.5)	25.2 (3.7)	25.0 (3.4)
Estimated FSIQ	100.8 (9.2)	101.4 (10.8)	100.2 (7.3)	100.9 (9.6)	101.4 (11.0)	100.4 (7.7)
Education (y)	12.8 (3.1)	12.8 (3.3)	12.7 (2.9)	12.7 (3.2)	12.7 (3.4)	12.7 (3.0)
Time from stroke to consent (months)	16.0 (17.6)	16.8 (22.6)	15.3 (12.0)	14.6 (12.1)	14.3 (12.8)	14.9 (11.8)
Modified Rankin score (0/1/2/3/4/5–6)	18/26/16/3/2/0	11/13/6/1/2/0	7/13/10/2/0/0	16/20/14/3/2/0	11/10/6/1/ 2/0	5/10/8/2/ 0/0
NIHSS (range 0–42)	1.0 [0.0, 2.0]	1.0 [0.0, 2.0]	1.0 [0.0, 2.0]	1.0 [0.0, 2.0]	0.5 [0.0, 2.0]	1.0 [0.0, 3.0]
WMH volume (mm^3^)	NA	NA	NA	14,600 [7200, 31,700]	11,800 [6800, 27,600]	15,700 [9200, 34,500]
Cerebral microbleeds, total count	1 [0, 4]	1 [0, 4]	1 [0, 4]	1 [0, 4]	1 [0, 4.5]	1.5 [0, 4]
SBP (mm Hg)	145 (16.6)	144 (16.4)	147 (17.1)	145 (16.6)	144 (14.8)	147 (18.7)
DBP (mm Hg)	81.0 (10.7)	81.0 (11.9)	81.0 (9.6)	79.9 (10.7)	80.5 (11.6)	79.2 (9.7)

Data are reported as mean (SD), except for modified Rankin score (number of participants with respective score listed), NIHSS score, WMH volume (both actual scores listed) and cerebral microbleed counts, which are reported as median [inter-quartile range]. Scoring on the MoCA ranges from 0 to 30, with a score of 26 or higher indicating normal cognitive ability. These scores have been adjusted for educational level (+1 if the participant had less than12 years of education). WMH volume and cerebral microbleed data are derived from post hoc analyses after trial completion. All other data are from the time of randomization.

*Abbreviations*. DBP: diastolic blood pressure; FSIQ: full scale intelligence quotient; MoCA: Montreal Cognitive Assessment; NIHSS: National Institutes of Health Stroke Scale; SBP systolic blood pressure; WMH: white matter hyperintensities.

The cohort of people who completed the protocol were older adults (15F/40 M, mean (SD) age 66.8 (8.6), range: 52–87 years, Table 1). They had a MOCA score of 25.1 (3.5) and 12.7 (3.2) years of education. In all cases, visit#1 took place at least six months after stroke or TIA. Visit#1 and visit#2 were 20 (19) days apart (mean (SD); range 7–117 days). Four participants completed visit#2 > 30 days after visit#1 (range 54–117 days).

There were no statistically significant treatment effects in any of the neuropsychological tests (Table 2). The “longest sequence” parameter for digit span forward trended an increase in performance (treatment effect 0.37, C.I. 0.01, 0.72; *P* = 0.0521). No such trend was apparent in the digit span backward (treatment effect −0.26, C.I. −0.64, 0.11; *P* = 0.176).

**Table 2:** Neuropsychological Test Data.

	First pre-dose score Mean (SD)	Change post Placebo Mean (SD)	Change post Tadalafil Mean (SD)	Treatment effect Mean (C.I.), P
CANTAB Choice Reaction time, seconds (*n* = 52)	463.2 (55.8)	−6.1 (27.9)	2.9 (36.6)	9.35 (−2.30, 20.99) 0.122
CANTAB Simple Reaction time, seconds (*n* = 52)	425.5 (93.0)	−10.0 (30.8)	−10.6 (79.2)	8.94 (−6.58, 24.45) 0.266
SOIP Adjusted	53.3 (17.4)	3.1 (8.5)	1.2 (11.2)	−2.11 (−5.67, 1.45) 0.250
SOIP Total	46.3 (14.1)	2.6 (6.6)	1.6 (6.7)	−1.11 (−3.40, 1.18) 0.348
SOIP Motor Speed Control Task (number correct)	40.5 (10.8)	2.5 (5.4)	1.7 (4.9)	−0.73 (−2.59, 1.13) 0.444
Digit span forward, longest digit	6.1 (1.4)	0.1 (1.1)	0.5 (1.3)	0.37 (0.01, 0.72) 0.0521
Digit span backward, longest digit (*n* = 54)	4.5 (1.4)	0.3 1.0)	0.0 (1.2)	−0.26 (−0.64, 0.11) 0.176
Semantic fluency	17.1 (4.9)	1.5 (4.1)	0.0 (4.2)	−0.43 (−2.72, 0.20) 0.568

Data for 55 non-withdrawn participants.

*Abbreviations*. CANTAB: Cambridge Neuropsychological Test Automated Battery; SOIP: speed of information processing.

There was no significant effect of group allocation (tadalafil at visit #1 and placebo at visit #2, or vice versa). No significant carry-over effect was detectable in any of the statistical models. There were no serious adverse reactions to the trial intervention. Adverse events are listed in Supplementary Table S1.

## 4 Discussion

This paper reports neuropsychological secondary outcomes from a double-blinded, randomized clinical trial of PDE5i treatment in older people with SVD. Single administration of tadalafil did not significantly change neuropsychological performance in the tests used here. These tests were selected to assess functionally relevant aspects of cognition, which have been implicated in SVD, and where four parallel forms were available. The issue of cognitive assessment in clinical trials for SVD, and for stroke in general, is an area of current debate [19]. There may be emerging cognitive instruments that will benefit future trials.

A trend towards a treatment effect on DSF was observed. DSF is typically considered highly stable, with negligible practice effects [20] and not amenable to short-term intervention. Some variation in DSF scores was observed in response to state and trait anxiety [21]. Treatment effects, when observed, typically result from extensive training, either in the form of repetitive practice or the development of elaborate metacognitive strategies (e.g. [22,23]) or a much larger number of administrations than in the present study. For example, 20 repeated daily administrations of common neuropsychological tests to people with or without brain injury, yielded performance increases on DSF only in the brain injury group and only in the latter 10 administrations [24]. Our data suggesting a potential treatment effect on DSF, a measure of attentional efficiency, a relatively low-level yet highly functionally relevant skill, and over a short time interval, are therefore striking. If confirmed in other studies, these findings suggest that the perception of DSF as a relatively immutable tool may need to be updated. Further, the effect sizes we report for DSF (and other cognitive measures) may be useful in planning future studies.

There was some sex imbalance in the trial. We do not know the reason for this. A recent meta-analysis reported a ratio of 1:1.67 women:men with SVD [25] but our ratio is much higher (1:2.42 among consented, 1:2.67 among completing participants, Table 1). As tadalafil is best known as a drug for enabling erectile function, we speculate that the sex bias might reflect more men than women being interested in participating in a tadalafil clinical trial.

The dose of tadalafil used here (20 mg) was within the range licensed for prescribing (5–40 mg) and between the dose typically prescribed in erectile dysfunction (5–10 mg) and that used in clinical trials for pulmonary arterial hypertension (40 mg). As described previously [17] plasma tadalafil concentrations were consistent with previous studies [8,9] and indicative of brain tadalafil concentrations well above the concentration required for half-maximal PDE5 inhibition.

PDE5 is expressed in brain tissue at mRNA and protein level [26,27]. As PDE5 is present in human brain neurons [6] effects of PDE5i on cognitive function might therefore be hypothesized [16]. In terms of acute PDE5i treatment, several groups have reported cognitive effects in rodents [11], [12], [13], [14], [15]. In non-human primates, acute treatment with sildenafil dose-dependently increased cognitive performance, in a paradigm considered a prefrontal task of executive function [28]. In humans, acute PDE5i treatment had little or no effect on cognitive performance in small cohorts of young healthy adults [29], healthy volunteers [30,31] or people with schizophrenia attending outpatient clinics [32]. These human findings accord with our present data from the PASTIS trial.

Longer term PDE5i treatment has been reported by several laboratories to produce cognitive changes in animals and humans. Mice and rats showed improved performance in standard behavioural assays following semi-chronic treatment (3–10 weeks) with sildenafil [11,[33], [34], [35]], vardenafil [36] or tadalafil [11,37]. In some studies, brain biochemical effects consistent with synaptic changes were detected. For example, treating mice for 21 days with sildenafil modified expression of synaptic proteins (synaptophysin, AMPA and NMDA receptors)[38]. Chronic treatment of aged Tg2576 mice with a novel agent, described as a dual antagonist of PDE5 and histone deacetylases, reduced cognitive deficits and enhanced dendritic spine density [33].

In human studies, two months of treatment with a PDE5i (udenafil) enhanced performance in the MMSE and a frontal assessment battery, in a small study of ED patients [39]. Similarly, in a small study of 12 patients with benign prostatic hyperplasia or erectile dysfunction, tadalafil gave some evidence of altered auditory evoked potentials and of cognitive improvement (in tests of mental processing speed and attention) [40]. By contrast, in a phase-2 randomised clinical trial in 70 ischaemic stroke survivors, 90-day treatment with a novel PDE5i did not change neuropsychological performance [41]. Nevertheless a recent systematic review concluded that there is a need for a clinical trial to test sildenafil for cognitive enhancement in AD [42]. This is further supported by a recent analysis of real-world prescribing data, where sildenafil and vardenafil were both in the top 20 medications associated with reduced dementia risk [43]. Overall, prior data suggest that long-term PDE5i treatment can lead to synaptic changes, consistent with enhanced cognition. We speculate that longer-term treatment with a brain-penetrant PDE5i might produce synaptic effects, leading to altered cognitive performance [16].

The present study has limitations. The cohort was small and was not powered *a priori* for detecting a change in neuropsychological performance. The PASTIS trial was designed to test for a change in brain blood flow, and was powered accordingly [17]. Most notably, the duration of treatment was brief, limited to one administration of tadalafil, with assessment only a few hours after dosing. In addition, the neuropsychological assessment was quite limited. The instruments used in this trial were focused on the key domains of interest, a comprehensive neuropsychological assessment was not attempted. Further, tadalafil is contra-indicated in patients with recent stroke or uncontrolled hypertension, hence there may be constraints on possible repurposing for use in SVD or dementia (Hainsworth et al., in press, [16]).

## 5 Conclusions

In conclusion, this study found insufficient evidence to support a significant difference between single dose tadalafil (20 mg) and placebo with respect to neuropsychological test performance. The trend observed on Digit Span Forward will serve to estimate effect sizes that may inform future studies.

## Funding

This study was joint-funded by UK Alzheimer's Society and Alzheimer's Drug Discovery Foundation (Grant Ref. 20140901, PI: AH Hainsworth). ABY Shtaya was supported by a National Institute for Health and Research Clinical Lectureship (CL-2015–16–001). The funding sources had no involvement in study design; in the collection, analysis and interpretation of data; in the writing of the report; and in the decision to submit the article for publication.

## Sponsor

The sponsor was St George's University of London (contact: sponsor@sgul.ac.uk).

## Declaration of Competing Interest

MMHP and LRB were employed as part of the PASTIS trial, JDI was Principal Investigator and AHH was Chief Investigator. CK is a PI on clinical trials funded by Bristol-Myers-Squibb and Bayer, and has received funding from NovoNordisk, all not relevant to the present trial. JDI has been a PI on clinical trials funded by Roche, Merck and Lupin Pharmaceuticals and has received funds from Nestle, Biogen and Roche, none relevant to the present trial. AHH leads MRC-Dementias Platform UK Vascular Experimental Medicine group. All other authors report no relevant disclosures. The trial was subject to an ICH-Good Clinical Practice (GCP) inspection by the UK medicines regulator, the MHRA, in September 2019, which identified a number of regulatory findings associated with the management of the trial. These are outlined in the supplementary information.

## Appendix Supplementary materials

Image, application 1

## References

[1] Kapasi A., DeCarli C., Schneider J.A. (2017). Impact of multiple pathologies on the threshold for clinically overt dementia. Acta Neuropathol..

[2] van Veluw S.J., Arfanakis K., Schneider J.A. (2022). Neuropathology of vascular brain health: insights from *ex vivo* magnetic resonance imaging-histopathology studies in cerebral small vessel disease. Stroke.

[3] Esiri M.M., Wilcock G.K., Morris J.H. (1997). Neuropathological assessment of the lesions of significance in vascular dementia. J. Neurol. Neurosurg. Psychiatry.

[4] Hainsworth A.H., Elahi F.M., Corriveau R.A. (2021). An introduction to therapeutic approaches to vascular cognitive impairment. Cereb. Circ. Cogn. Behav..

[5] Vasita E., Yasmeen S., Andoh J., Bridges L.R., Kruuse C., Pauls M.M.H., Pereira A.C., Hainsworth A.H. (2019). The cGMP-degrading enzyme phosphodiesterase-5 (PDE5) in cerebral small arteries of older people. J. Neuropathol. Exp. Neurol..

[6] Teich A.F., Sakurai M., Patel M., Holman C., Saeed F., Fiorito J., Arancio O. (2016). PDE5 exists in human neurons and is a viable therapeutic target for neurologic disease. J. Alzheimers Dis..

[7] Pauls M.M.H., Clarke N., Trippier S., Betteridge S., Howe F.A., Khan U., Kruuse C., Madigan J.B., Moynihan B., Pereira A.C., Rolfe D., Rostrup E., Haig C.E., Barrick T.R., Isaacs J.D., Hainsworth A.H. (2017). Perfusion by arterial spin labelling following single dose tadalafil in small vessel disease (PASTIS): study protocol for a randomised controlled trial. Trials.

[8] Forgue S.T., Patterson B.E., Bedding A.W., Payne C.D., Phillips D.L., Wrishko R.E., Mitchell M.I. (2006). Tadalafil pharmacokinetics in healthy subjects. Br. J. Clin. Pharmacol..

[9] Forgue S.T., Phillips D.L., Bedding A.W., Payne C.D., Jewell H., Patterson B.E., Wrishko R.E., Mitchell M.I. (2007). Effects of gender, age, diabetes mellitus and renal and hepatic impairment on tadalafil pharmacokinetics. Br. J. Clin. Pharmacol..

[10] Garcia-Osta A., Cuadrado-Tejedor M., Garcia-Barroso C., Oyarzabal J., Franco R. (2012). Phosphodiesterases as therapeutic targets for Alzheimer's disease. ACS Chem. Neurosci..

[11] Garcia-Barroso C., Ricobaraza A., Pascual-Lucas M., Unceta N., Rico A.J., Goicolea M.A., Salles J., Lanciego J.L., Oyarzabal J., Franco R., Cuadrado-Tejedor M., Garcia-Osta A. (2013). Tadalafil crosses the blood-brain barrier and reverses cognitive dysfunction in a mouse model of AD. Neuropharmacology.

[12] Garcia-Barroso C., Ugarte A., Martinez M., Rico A.J., Lanciego J.L., Franco R., Oyarzabal J., Cuadrado-Tejedor M., Garcia-Osta A. (2014). Phosphodiesterase inhibition in cognitive decline. J. Alzheimers Dis..

[13] Argyrousi E.K., Heckman P.R., van Hagen B.T., Muysers H., van Goethem N.P., Prickaerts J. (2020). Pro-cognitive effect of upregulating cyclic guanosine monophosphate signalling during memory acquisition or early consolidation is mediated by increased AMPA receptor trafficking. J. Psychopharmacol..

[14] Rodefer J.S., Saland S.K., Eckrich S.J. (2012). Selective phosphodiesterase inhibitors improve performance on the ED/ID cognitive task in rats. Neuropharmacology.

[15] Devan B.D., Bowker J.L., Duffy K.B., Bharati I.S., Jimenez M., Sierra-Mercado D., Nelson C.M., Spangler E.L., Ingram D.K. (2006). Phosphodiesterase inhibition by sildenafil citrate attenuates a maze learning impairment in rats induced by nitric oxide synthase inhibition. Psychopharmacology.

[16] Hainsworth A.H., Arancio O., Elahi F.M., Isaacs J.D., Cheng F. (2023). PDE5 inhibitor drugs for use in dementia?. Alzheimer's Dement. Transl. Res. Clin. Interv..

[17] Pauls M.M.H., Binnie L.R., Benjamin P., Betteridge S., Clarke B., Dhillon M.K., Ghatala R., Hainsworth F.A.H., Howe F.A., Khan U., Kruuse C., Madigan J.B., Moynihan B., Patel B., Pereira A.C., Rostrup E., Shtaya A.B.Y., Spilling C.A., Trippier S., Williams R., Young R., Barrick T.R., Isaacs J.D., Hainsworth A.H. (2022). The PASTIS trial: testing tadalafil for possible use in vascular cognitive impairment. Alzheimers Dement..

[18] Smith E.E., Markus H.S. (2020). New treatment approaches to modify the course of cerebral small vessel diseases. Stroke.

[19] Quinn T.J., Richard E., Teuschl Y., Gattringer T., Hafdi M., O’Brien J.T., Merriman N., Gillebert C., Huygelier H., Verdelho A., Schmidt R., Ghaziani E., Forchammer H., Pendlebury S.T., Bruffaerts R., Mijajlovic M., Drozdowska B.A., Ball E., Markus H.S. (2021). European Stroke Organisation and European Academy of Neurology joint guidelines on post-stroke cognitive impairment. Eur. J. Neurol..

[20] Lezak D.B., Bigler E.D., Tranel D. (2012).

[21] Nyberg J., Henriksson M., Wall A., Vestberg T., Westerlund M., Walser M., Eggertsen R., Danielsson L., Kuhn H.G., Aberg N.D., Waern M., Aberg M. (2021). Anxiety severity and cognitive function in primary care patients with anxiety disorder: a cross-sectional study. BMC Psychiatry.

[22] Cavallini E., Pagnin A., Vecchi T. (2003). Aging and everyday memory: the beneficial effect of memory training. Arch. Gerontol. Geriatr..

[23] Shipstead Z., Redick T.S., Engle R.W. (2012). Is working memory training effective?. Psychol. Bull..

[24] Wilson B.A., Watson P.C., Baddeley A.D., Emslie H., Evans J.J. (2000). Improvement or simply practice? The effects of twenty repeated assessments on people with and without brain injury. J. Int. Neuropsychol. Soc..

[25] Jimenez-Sanchez L., Hamilton O.K.L., Clancy U., Backhouse E.V., Stewart C.R., Stringer M.S., Doubal F.N., Wardlaw J.M. (2021). Sex differences in cerebral small vessel disease: a systematic review and meta-analysis. Front. Neurol..

[26] Sanderson T.M., Sher E. (2013). The role of phosphodiesterases in hippocampal synaptic plasticity. Neuropharmacology.

[27] Lakics V., Karran E.H., Boess F.G. (2010). Quantitative comparison of phosphodiesterase mRNA distribution in human brain and peripheral tissues. Neuropharmacology.

[28] Rutten K., Van Donkelaar E.L., Ferrington L., Blokland A., Bollen E., Steinbusch H.W., Kelly P.A., Prickaerts J.H. (2009). Phosphodiesterase inhibitors enhance object memory independent of cerebral blood flow and glucose utilization in rats. Neuropsychopharmacology.

[29] Reneerkens O.A., Sambeth A., Van Duinen M.A., Blokland A., Steinbusch H.W., Prickaerts J. (2013). The PDE5 inhibitor vardenafil does not affect auditory sensory gating in rats and humans. Psychopharmacology.

[30] Schultheiss D., Muller S.V., Nager W., Stief C.G., Schlote N., Jonas U., Asvestis C., Johannes S., Munte T.F. (2001). Central effects of sildenafil (Viagra) on auditory selective attention and verbal recognition memory in humans: a study with event-related brain potentials. World J. Urol..

[31] Grass H., Klotz T., Fathian-Sabet B., Berghaus G., Engelmann U., Kaferstein H. (2001). Sildenafil (Viagra): is there an influence on psychological performance?. Int. Urol. Nephrol..

[32] Goff D.C., Cather C., Freudenreich O., Henderson D.C., Evins A.E., Culhane M.A., Walsh J.P. (2009). A placebo-controlled study of sildenafil effects on cognition in schizophrenia. Psychopharmacology.

[33] Cuadrado-Tejedor M., Hervias I., Ricobaraza A., Puerta E., Perez-Roldan J.M., Garcia-Barroso C., Franco R., Aguirre N., Garcia-Osta A. (2011). Sildenafil restores cognitive function without affecting beta-amyloid burden in a mouse model of Alzheimer's disease. Br. J. Pharmacol..

[34] Venkat P., Chopp M., Zacharek A., Cui C., Landschoot-Ward J., Qian Y., Chen Z., Chen J. (2019). Sildenafil treatment of vascular dementia in aged rats. Neurochem. Int..

[35] Orejana L., Barros-Minones L., Jordan J., Puerta E., Aguirre N. (2012). Sildenafil ameliorates cognitive deficits and tau pathology in a senescence-accelerated mouse model. Neurobiol. Aging.

[36] Gulisano W., Tropea M.R., Arancio O., Palmeri A., Puzzo D. (2018). Sub-efficacious doses of phosphodiesterase 4 and 5 inhibitors improve memory in a mouse model of Alzheimer's disease. Neuropharmacology.

[37] Al-Amin M.M., Hasan S.M., Alam T., Hasan A.T., Hossain I., Didar R.R., Alam M.A., Rahman M.M. (2014). Tadalafil enhances working memory, and reduces hippocampal oxidative stress in both young and aged mice. Eur. J. Pharmacol..

[38] Araujo S., Duarte-Silva E., Marinho C.G.S., Oliveira W.H., Franca M.E.R., Los D., Peron G., Tomaz L., Bonfanti A.P., Verinaud L., Peixoto C.A. (2020). Effect of sildenafil on neuroinflammation and synaptic plasticity pathways in experimental autoimmune encephalomyelitis. Int. Immunopharmacol..

[39] Shim Y.S., Pae C.U., Kim S.W., Kim H.W., Kim J.C., Koh J.S. (2011). Effects of repeated dosing with Udenafil (Zydena) on cognition, somatization and erection in patients with erectile dysfunction: a pilot study. Int. J. Impot. Res..

[40] Urios A., Ordono F., Garcia-Garcia R., Mangas-Losada A., Leone P., Gallego J.J., Cabrera-Pastor A., Megias J., Ordono J.F., Felipo V., Montoliu C. (2019). Tadalafil treatment improves inflammation, cognitive function, and mismatch negativity of patients with low urinary tract symptoms and erectile dysfunction. Sci. Rep..

[41] Di Cesare F., Mancuso J., Woodward P., Bednar M.M., Loudon P.T., Group A.S.S. (2016). Phosphodiesterase-5 inhibitor PF-03049423 effect on stroke recovery: a double-blind, placebo-controlled randomized clinical trial. J. Stroke Cerebrovasc. Dis..

[42] Sanders O. (2020). Sildenafil for the treatment of Alzheimer's disease: a systematic review. J. Alzheimers Dis. Rep..

[43] Fang J., Zhang P., Zhou Y., Chiang C., Tan J., Hou Y., Stauffer S., Li L., Pieper A., Cummings J., Cheng F. (2021). Endophenotype-based in silico network medicine discovery combined with insurance record data mining identifies sildenafil as a candidate drug for Alzheimer's disease. Nat. Aging.

