# Supplementary material for: Testing the cognitive effects of tadalafil. Neuropsychological secondary outcomes from the PASTIS trial
Source: Cereb Circ Cogn Behav. 2023 Sep 26;5:100187. doi: 10.1016/j.cccb.2023.100187 (PMC10550803; doi:10.1016/j.cccb.2023.100187)
Supplement: Supplementary file 1 [file mmc1.pdf]

Testing the cognitive effects of tadalafil. Neuropsychological secondary outcomes from the PASTIS trial: Supplemental File

Mathilde MH Pauls[1,2], Jessica Fish[3,4], Lauren R Binnie[1], Philip Benjamin[1,5], Shai Betteridge[3], Brian Clarke[2], Mohani-Preet K Dhillon[1], Rita Ghatala[6], Fearghal AH Hainsworth[1], Franklyn A Howe[1], Usman Khan[2], Christina Kruuse[7], Jeremy B Madigan[5], Barry Moynihan[2,8], Bhavini Patel[2], Anthony C Pereira[1,2], Egill Rostrup[9], Anan BY Shtaya[1], Catherine A Spilling[1], Sarah Trippier[6], Rebecca Williams[6], Robin Young[10], Thomas R Barrick[1], Jeremy D Isaacs[1,2] and Atticus H Hainsworth[1,2 *]

[1]Molecular & Clinical Sciences Research Institute, St George's University of London, UK; [2]Department of Neurology, St George's University Hospitals NHS Foundation Trust, London, UK;[3]Neuropsychology, St George's University Hospitals NHS Foundation Trust, London, UK; [4]School of Health & Wellbeing, University of Glasgow, UK. [5]Neuroradiology, St George's University Hospitals NHS Foundation Trust, London, UK; [6]South London Stroke Research Network, London, UK; [7]Department of Neurology and Neurovascular Research Unit, Herlev Gentofte Hospital, Denmark; [8]Department of Medicine, Royal College of Surgeons in Ireland, Beaumont Hospital, Dublin, Ireland; [9]Mental Health Centre, University of Copenhagen, Glostrup, Denmark; [10]Robertson Centre for Biostatistics, University of Glasgow, UK.

*correspondence: Dr AH Hainsworth PhD, St George's University of London, Cranmer Terrace, London SW17 0RE, United Kingdom. Tel:+442087255586 fax:+442087252950. email: ahainsworth@sgul.ac.uk

Supplementary Methods

Changes to Methods after trial commencement

Amendments were made to the published protocol [1]:

- to perform some cognitive testing in Visit 0 (from 02 September 2015).
- Eligibility criteria were adjusted to allow lower age limit of 50 and lower Creatinine Clearance of 30ml/min (from 29 October 2015).

Inclusion and Exclusion criteria

Inclusion criteria were as follows. 1, radiological evidence of SVD, defined as: MRI evidence of lacunar infarct(s) up to 15 mm maximum diameter and/or confluent deep WMH (grade 2 or higher on the Fazekas scale [2]). 2, clinical evidence of SVD defined as either: lacunar stroke syndrome with symptoms lasting at least 24 hours, occurring at least 6 months prior to visit#1; or: transient ischaemic attack lasting <24 hours with limb weakness, hemi-sensory loss or dysarthria at least 6 months previously and with diffusion-weighted MRI performed acutely showing lacunar infarction. If MRI was not performed within 10 days of transient ischaemic attack, a lacunar infarction in an anatomically appropriate position as demonstrated on a subsequent MRI was also deemed eligible. 3, age at least 50 years. 4, imaging of the carotid arteries in the previous 12 months, demonstrating less than 70% stenosis in both internal carotid arteries or less than 50% stenosis in both internal carotids if measured in previous 12-60 months. Exclusion criteria included: known diagnosis of dementia; cortical infarction (more than 15 mm maximum width); SBP<90 mmHg; DBP<50 mmHg; creatinine clearance <30ml/min; stroke or TIA within the previous 6 months; concomitant use of PDE5i. A full list of exclusion criteria is given in the published protocol [1].

Baseline Cognitive tests

The Montreal Cognitive Assessment (MOCA) [3]. This brief and widely-used cognitive screening tool produces a total score, based on sub-task scores in the domains of orientation, visuospatial/executive, naming, attention, language, and memory recall. A cut-off score of 26 for normal function is typically used, and total scores are reported here.

The Test of Premorbid Function (TOPF) [4] is an oral reading test using single words with irregular grapheme-phoneme relationships. As this is a crystallised cognitive ability, the number of words correctly pronounced can be used to estimate optimal intellectual ability, and in particular estimated WAIS-IV Index Scores. Here we report the estimated full-scale IQ, (FSIQ).

Neuropsychological tests at study visits

CANTAB® Reaction Time subtest (CANTAB® cognitive assessment software, Cambridge Cognition (2019). All rights reserved. www.cantab.com). In this computerised task the participant must make a motor response, as rapidly as possible, to a visual stimulus appearing in either a single location (simple RT) or one of five locations (choice RT). This test indexes alertness and motor response speed. The parameters used in the analysis were mean scores for simple and choice-format RTs.

Speed of Information Processing (SOIP) subtest of the BMIPB (Brain Injury Rehabilitation Trust Memory and Information Processing Battery; website: BIRT Memory and Information Processing Battery (abdn.ac.uk)). In this pen-and-paper task participants view a series of number sequences, each comprising five 2-digit numbers, and must cross a line through the second-highest number in that sequence. They must work through the sequences as quickly as possible whilst avoiding errors, over four minutes. A motor speed control condition of the task is also completed, in which the number arrays are replaced by arrays each containing four dash symbols and one number eleven (e.g. "- - 11 - -"). The participant must draw a line through as many '11s' as possible within 25 seconds. The parameters analysed were SOIP Adjusted score (i.e. information processing speed score after taking motor speed into account), SOIP Total score (number of correct cancellations in the numerical condition) and SOIP Speed (number of correct cancellations in the motor control condition).

Digit Span Forward (DSF; Repeatable Battery for the Assessment of Neuropsychological Status, RBANS[5], updated[6]. In this task participants listen to steadily-paced (1 per second) single digit sequences of increasing length, and repeat them verbatim. DSF is considered to index attentional efficiency and 'freedom from distractibility' [7].

Digit Span Backward. This test also requires participants to listed to digit sequences of increasing length, but they participant must repeat them in the reverse order than that presented (i.e., digits backward). Its cognitive demands are different than those required for the DSF task, being more effortful and requiring mental double tracking. Hence Digit Span Backward is best thought of as an index of verbal working memory. The RBANS Digit Span subtest involves administration of two strings per span length, starting with 2-digit strings and increasing to 9-digit strings, and has no reverse DSB component. For this study, to obtain measures of both forward and backward span, only one trial was administered at each length, with the RBANS first string list used for DSF and the secon string list used for DSB. The parameters analysed were 'longest sequence DSF' and 'longest sequence DSB', denoting the number of items in the longest accurately recalled string.

Semantic fluency (RBANS) [5]. In this task participants are required to say as many exemplars of words belonging to a set category (e.g. animals) as possible within 60 seconds. This measures verbal productivity and fluency, and the parameter analysed was total words generated.

Blood sampling and analyses

At the end of each study visit, and at least 3 h post dosing, a blood sample (approximately 5 ml) was taken for full blood count. A second blood sample was taken (5 ml) for subsequent analysis of tadalafil concentration. Blood was taken in purple capped EDTA tubes, inverted to mix, and centrifuged at room temperature at 3000 RPM for 5 minutes to remove cellular material. Plasma (approximately 1.5-2.0 ml) was decanted into a labelled plastic cryovial, then transferred to a designated -80 ^{o}C freezer. Plasma tadalafil concentration was measured by LC-MS-MS assay (ASI Bioanalytics Ltd, London UK, https://www.bioanalytics.co.uk/).

The trial was subject to an ICH-Good Clinical Practice inspection by the Medicines and Healthcare Products Regulatory Agency (MHRA), the statutory regulator in the UK in September 2019 which identified breaches of ICH-GCP associated with sample analysis. The analytical method used was a forensic toxicology procedure rather than a method which had been validated against the European Medicines Agency Bioanalytical Method Validation guidance. Though tadalafil has high freeze/thaw stability [8] the impact of the storage of samples and freeze/thaw cycles, along with other assessments stated in the European Medicines Agency guidance, on tadalafil plasma concentration were not determined during this trial. All tadalafil concentrations reported here were derived from first analysis, so were not subject to repeated freeze/thaw effects. The maximum duration between sample storage at -80 ^{o}C and analysis was 892 days (median 454 days, IQR 366-586 days).

An additional ICH-GCP breach identified that plasma tadalafil levels were analysed prematurely in fifteen participants, resulting in the chief investigator (AHH) being unblinded to the treatment group for these individuals. The trial had been designed as a double-blinded randomized control trial which meant that no members of the research team should have been aware of IMP regime of any of the subjects during the trial. As the chief investigator had no direct role in patient assessment or data acquisition and performed none of the data analyses reported, this was not considered to have compromised the trial outcomes or conclusions and the study continues to remain double-blinded (i.e. patient-blinded and clinician-blinded).

Supplemental Table S1. Adverse Events in the PASTIS trial

Partici-pant No.	Age (y)	Sex	Group 1 or 2	Treatment at onset of AE	Duration of AE	Description of AE
6	75	f	2	Placebo	2 d	Headache and vomited after visit 1 at home; had passed by the next morning. Withdrew from the trial.
13	72	m	2	Placebo	2 d	Had a cold and did not tolerate first MRI scan, study visit abandoned.
15	61	f	1	Placebo	1 day	Had headache lasting 2 mins after lunch.
17	69	f	2	Placebo	1 day	Diabetes mellitus type 1 (all adult life); had a hypoglycaemic event during visit 1. Resolved after a sugary drink and fruit.
22	77	f	1	Placebo	6 days	Had a chest infection treated by GP with antibiotics between visits 1 and 2.
33	59	f	2	Tadalafil	5 days	COPD Asthma (lifelong); had lower respiratory tract infection.
33	59	f	2	Tadalafil	3 days	Left knee pain (psoriatric arthritis).
37	73	f	1	Placebo	11 days	Sore throat, cough and feeling unwell. Cancelled visit 2 due to inability to lie still with cough. Re-enrolled as #51.
41	56	m	2	Placebo	1 day	Felt flushed and slightly faint for 5 mins, starting about 30 mins after treatment. Recovered spontaneously and felt better after a few hours.

49	57	m	2	Placebo	1 day	Panic attack in MRI scanner during visit 1. Withdrew from the trial.
59	72	f	1	Tadalafil	1 day	Felt lightheaded after first MRI on visit 1. Had a sandwich, felt better in 10 mins.

Abbreviations: AE: adverse event. Note that two AEs relate to the same participant (#33).

Supplementary References

[1] M.M.H. Pauls, N. Clarke, S. Trippier, S. Betteridge, F.A. Howe, U. Khan, C. Kruuse, J.B. Madigan, B. Moynihan, A.C. Pereira, D. Rolfe, E. Rostrup, C.E. Haig, T.R. Barrick, J.D. Isaacs, A.H. Hainsworth. Perfusion by Arterial Spin labelling following Single dose Tadalafil In Small vessel disease (PASTIS): study protocol for a randomised controlled trial. Trials. 2017;18.

[2] L.O. Wahlund, F. Barkhof, F. Fazekas, L. Bronge, M. Augustin, M. Sjogren, A. Wallin, H. Ader, D. Leys, L. Pantoni, F. Pasquier, T. Erkinjuntti, P. Scheltens, C. European Task Force on Age-Related White Matter. A new rating scale for age-related white matter changes applicable to MRI and CT. Stroke. 2001;32:1318-1322.

[3] Z.S. Nasreddine, N.A. Phillips, V. Bedirian, S. Charbonneau, V. Whitehead, I. Collin, J.L. Cummings, H. Chertkow. The Montreal Cognitive Assessment, MoCA: a brief screening tool for mild cognitive impairment. J Am Geriatr Soc. 2005;53:695-699.

[4] D. Wechsler. *Test of Premorbid Functioning – UK Version (TOPF).* London, UK: Pearson; 2011.

[5] C. Randolph, M.C. Tierney, E. Mohr, T.N. Chase. The Repeatable Battery for the Assessment of Neuropsychological Status (RBANS): preliminary clinical validity. J Clin Exp Neuropsychol. 1998;20:310-319.

[6] C. Randolph. *Repeatable battery for the assessment of neuropsychological status (RBANS): Update.* . London, UK: Pearson; 2012.

[7] M.D.H. Lezak, D.B.; Bigler,E.D.; Tranel,D. *Neuropsychological assessment* Oxford, UK: OUP; 2012.

[8] K.Y. Rust, H. Wilkens, R. Kaiser, D. Bregel, J. Wilske, T. Kraemer. Detection and validated quantification of the phosphodiesterase type 5 inhibitors sildenafil, vardenafil, tadalafil, and 2 of their metabolites in human blood plasma by LC-MS/MS--application to forensic and therapeutic drug monitoring cases. Ther Drug Monit. 2012;34:729-735.
